# Baseline characteristics of SARS-CoV-2 vaccine non-responders in a large population-based sample

**DOI:** 10.1371/journal.pone.0303420

**Published:** 2024-05-13

**Authors:** Ashraf Yaseen, Stacia M. DeSantis, Rachit Sabharwal, Yashar Talebi, Michael D. Swartz, Shiming Zhang, Luis Leon Novelo, Cesar L. Pinzon-Gomez, Sarah E. Messiah, Melissa Valerio-Shewmaker, Harold W. Kohl, Jessica Ross, David Lakey, Jennifer A. Shuford, Stephen J. Pont, Eric Boerwinkle

**Affiliations:** 1 The University of Texas Health Science Center at Houston, School of Public Health in Houston, Houston, TX, United States of America; 2 The University of Texas Health Science Center at Houston, School of Public Health in Dallas, Dallas, TX, United States of America; 3 Center for Pediatric Population Health, UTHealth School of Public Health, Dallas, Texas, United States of America; 4 The University of Texas Health Science Center at Houston, School of Public Health in Brownville, Brownsville, TX, United States of America; 5 The University of Texas Health Science Center at Houston, School of Public Health in Austin, Austin, TX, United States of America; 6 University of Texas at Austin, Austin, TX, United States of America; 7 University of Texas System, Austin, TX, United States of America; 8 The University of Texas Health Science Center Tyler, Tyler, TX, United States of America; 9 Texas Department of State Health Services, Austin, TX, United States of America; University of Ilorin, NIGERIA

## Abstract

**Introduction:**

Studies indicate that individuals with chronic conditions and specific baseline characteristics may not mount a robust humoral antibody response to SARS-CoV-2 vaccines. In this paper, we used data from the Texas Coronavirus Antibody REsponse Survey (Texas CARES), a longitudinal state-wide seroprevalence program that has enrolled more than 90,000 participants, to evaluate the role of chronic diseases as the potential risk factors of non-response to SARS-CoV-2 vaccines in a large epidemiologic cohort.

**Methods:**

A participant needed to complete an online survey and a blood draw to test for SARS-CoV-2 circulating plasma antibodies at four-time points spaced at least three months apart. Chronic disease predictors of vaccine non-response are evaluated using logistic regression with non-response as the outcome and each chronic disease + age as the predictors.

**Results:**

As of April 24, 2023, 18,240 participants met the inclusion criteria; 0.58% (N = 105) of these are non-responders. Adjusting for age, our results show that participants with self-reported immunocompromised status, kidney disease, cancer, and “other” non-specified comorbidity were 15.43, 5.11, 2.59, and 3.13 times more likely to fail to mount a complete response to a vaccine, respectively. Furthermore, having two or more chronic diseases doubled the prevalence of non-response.

**Conclusion:**

Consistent with smaller targeted studies, a large epidemiologic cohort bears the same conclusion and demonstrates immunocompromised, cancer, kidney disease, and the number of diseases are associated with vaccine non-response. This study suggests that those individuals, with chronic diseases with the potential to affect their immune system response, may need increased doses or repeated doses of COVID-19 vaccines to develop a protective antibody level.

## Introduction

Individuals with certain malignancies [1,2], organ transplant recipients [3–5], and immune-compromised individuals [5–7] could mount either an incomplete or sub-optimal antibody response to “primary series vaccination”, or PSV. PSV in the United States is defined as receiving two mRNA vaccines (Moderna or Pfizer) or one non-mRNA vaccine (Johnson & Johnson or Novavax) [2–4]. Agha M et al. [1] found that 46% of hematologic malignancy patients did not produce antibodies following SARS-CoV-2 mRNA vaccination and were therefore vaccine non-responders. Zeng C et al. [2] studied the neutralizing antibody response in 160 cancer patients diagnosed with chronic lymphocytic leukemia, lung cancer, breast cancer, and various non-Hodgkin’s lymphomas after receiving two mRNA vaccine doses. They discovered that cancer patients exhibited reduced neutralizing antibody titer compared to 46 mRNA-vaccinated healthcare providers who served as healthy controls. Boyarsky BJ et al. [3,4] presented a study on the antibody response to SARS-CoV-2 mRNA vaccines in solid organ transplant recipients. Their study included 658 transplant recipients who received two doses of SARS-CoV-2 mRNA vaccine. Their results demonstrated a low response rate among the study subjects. Ferri C et al. [6] presented an observational study to evaluate COVID-19 vaccines in patients with autoimmune systemic diseases (ASD). The study included 478 ASD patients and 502 individuals as the control group. Their results show a higher percentage of non-responders to vaccine in ASD patients compared to controls [13.2% vs. 2.8%; p < 0.0001]. Galmiche S et al. [7] reviewed the literature to assess the immunogenicity, efficacy, and effectiveness of COVID-19 vaccines in immunocompromised populations. They found that non-response is higher among solid organ transplant recipients (range 18–100%) and patients with hematological malignancy (range 14–61%) and lower in patients with cancer (range 2–36%) and patients on dialysis (range 2–30%).

Non-response can occur for any vaccine (including SARS-CoV-2) and is influenced by various factors, including age, health status, and genetics [8–10]. Management strategies for non-responders include more frequent booster shots for COVID-19 and/or higher doses of vaccines. Previous studies have shown that non-response to COVID-19 vaccines can vary depending on factors such as age and vaccine type but generally is lower than non-response rates for some standard vaccines. Specifically, the measles, mumps, rubella (MMR), and inactivated polio vaccines (IPV) have very low non-response rates, with most recipients developing protective immunity. The hepatitis B vaccine typically has a low non-response rate, with most individuals mounting a sufficient immune response after the recommended doses [11]. However, it’s crucial to note that non-response definitions can vary among vaccines, and the landscape may have evolved since 2021 due to factors like emerging variants and ongoing research, making it essential to consult current sources for the latest non-response data.

Identifying predictors of vaccine non-response to SARS-CoV-2 is urgently needed as the COVID-19 pandemic enters its fourth year. The emergence of variants and inconsistencies in vaccine coverage have resulted in a continued public health threat. This manuscript leverages a large cohort to expand on smaller clinical studies regarding conditions resulting in higher vaccine non-response odds. To our knowledge, an extensive epidemiologic cohort survey confirming these findings at the population level has yet to be presented.

## Materials and methods

### Study design and settings

Since October 2020, the Texas Coronavirus Antibody REsponse Survey (Texas CARES) program has enrolled more than 90,000 participants ages 5-to-90 years old in a state-wide antibody prevalence surveillance program to inform stakeholders about the human antibody response to SARS-CoV-2 and its vaccines, the longevity of immune response, and how this response varies in a demographically diverse state-wide population. The recruitment of participants began on September 1, 2020 (as a Pilot study). This project is still ongoing, but enrollment of new participants stopped on December 4, 2023. The program has been described in detail previously [12].

Briefly, participants include Federally Qualified Health Centers (FQHC) patients and staff, schoolteachers, children, retail workers, university students, and unemployed individuals ages 5-to-90 years across the state of Texas. The protocol includes completion of (i) 4 longitudinal blood draws for nucleocapsid and spike protein antibody assessment; (ii) a continually updated online questionnaire capturing information on social factors, COVID-19 illness and symptom history, post-COVID conditions, health behaviors, adherence to community mitigation strategies, and pre-existing chronic diseases (including immunocompromised state). The data collected through the survey, including chronic diseases, are self-reported.

The large number of participants affords the ability to ascertain the most prevalent chronic diseases in the uncommon event of vaccine non-response to the most prevalent vaccines in the US (2 doses of Pfizer-BioNTech or Moderna’s mRNA vaccine or one dose of Johnson & Johnson or Novavax viral vector vaccine), to determine which chronic diseases were most associated with vaccine non-response.

Previous reports on the kinetics of these mRNA and viral vector vaccines show that between 2 to 4 weeks after complete vaccination, there is a peak in antibody response that progressively decreases between 6 to 8 months. The reported difference is that the viral vector vaccine initially induces lower antibody responses but is stable for up to 8 months [13].

Nucleocapsid antibody status was assessed using the Roche Elecsys® Anti-SARS-CoV-2 Immunoassay (N test). The Roche assay detects high-affinity antibodies to SARS-CoV-2 using a modified recombinant protein representing the nucleocapsid (N) antigen to determine SARS-CoV-2 antibodies [14]. The test has a published sensitivity of 99.5% (95% CI: 97.0–100) and 99.8% specificity (95% CI: 99.69–99.88) in diagnostic specimens (n = 2861). Vaccination response status was assessed via the Roche Elecsys® Anti-SARS-CoV-2 S Immunoassay (S test). The S test has a published positive percent agreement (PPA) of 96.6% (95% CI: 93.4–98.5) in clinical samples at least 15 days after infection and 100% overall specificity in diagnostic specimens (n = 1468) [15]. Immunoglobulin G (IgG) levels measured by ElectroChemiLuminiscence (ECL) serology assays have been shown to correlate with neutralization assay [16], and IgG anti-Spike antibodies have been highly correlated with ID50 neutralization in a validated pseudoviral assay and with vaccine efficacies against infection with different variants of the SARS-CoV-2 virus [17]. Furthermore, measuring antibodies to spike protein is widely available and can be done relatively inexpensively, making it ideal for testing large numbers of people, as we have done in this longitudinal serosurvey.

### Statement of ethical approval

All protocols were reviewed and approved by the University of Texas Health Science Center, Committee for the Protection of Human Subjects, but also deemed public health practice by the Texas Department of State Health Services IRB. All adults consented electronically, and parents or designated caregivers provided (electronic) proxy informed consent for children and adolescents (under 18 years) to participate in the Texas CARES study. Adolescents over the age of 12 had the option to sign assent and complete the questionnaire. No adolescents refused to provide assent or participate. The informed consent process was seamlessly integrated with the online questionnaire to ease the burden on respondents and maximize survey completion.

### Cohort inclusion criteria

**Fig 1:**
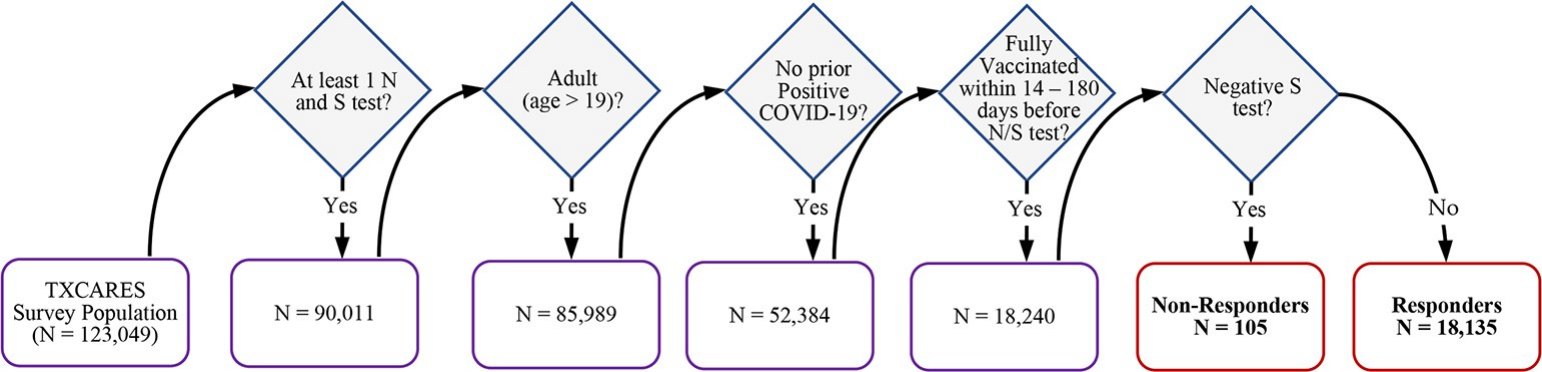
Cohort inclusion criteria.

The study cohort includes adult participants (age>19) who had at least one valid nucleocapsid and spike protein test on record, without prior COVID-19 infection (reported by the survey and confirmed by the negative Roche N test immunoassay), and reported being fully vaccinated within 14–180 days prior to their spike protein assays. Vaccine non-responders were those individuals in the study cohort who tested negative for the Roche S test after being fully vaccinated. Fig 1 presents a step-by-step flow chart of inclusion criteria with the number of participants at each step.

### Statistical methods/data analysis

Demographic and health predictors of non-response are presented using frequencies (N) and percentages (%). The preliminary statistical analysis suggested *Age* as a significant factor in our models (S1 Fig). As a result, chronic disease predictors of vaccine non-response are evaluated using age-adjusted logistic regression with non-response (vs. response) as the outcome and each disease + *age* as the predictors. Furthermore, the change in proportions of Spike antibody serostatus was evaluated over four time-points. Frequencies and percentages of reported diseases by responder status are presented along with *age*-adjusted odds ratios and 95% confidence intervals (OR [95% CI]). Change in the response status was then examined following vaccine booster doses for those individuals who reported getting boosters. A breakdown of some of these non-responders is presented in the (S2 and S3 Figs).

## Results

As of April 24, 2023, a total of 18,240 participants met the inclusion criteria for this survey; 0.58% (N = 105) of these were non-responders.

The reported demographic and health composition of the cohort, shown in Table 1, is as follows: 66.4% are female; 75.3% are non-Hispanic White, 1.9% are non-Hispanic Black, 12.2% are Hispanic, 5.7% are Asian, 4.9% report other or missing for race/ethnicity; 38.3% have healthy body mass index while 59% are overweight or obese; 92.8% received an mRNA vaccine, 6.8% received the Johnson and Johnson vaccine. Finally, 53.8% report at least one chronic disease. Please note that in Table 1, BMI was calculated from the participants’ self-reported height and weight and then categorized according to the CDC guidelines for BMI into (Healthy, Obesity, Overweight, and Underweight). The Pearson’s Chi-squared tests in Table 1 suggest a statistically significant relationship between COVID-19 vaccination non-response and the covariates of age, vaccine type, and chronic disease (both the presence and overall number).

**Table 1:** Demographic and health characteristics by vaccine response status.

	Total (N = 18240)	Non-Responder (N = 105)	Responder (N = 18135)	P-values*
**Age**				< 0.001
20–29	1011 (5.5%)	4 (3.8%)	1007 (5.6%)	
30–39	3258 (17.9%)	10 (9.5%)	3248 (17.9%)	
40–49	4058 (22.2%)	19 (18.1%)	4039 (22.3%)	
50–64	6598 (36.2%)	37 (35.2%)	6561 (36.2%)	
65–74	2721 (14.9%)	26 (24.8%)	2695 (14.9%)	
75+	594 (3.3%)	9 (8.6%)	585 (3.2%)	
**Gender**				0.96**
Female	12105 (66.4%)	69 (65.7%)	12036 (66.4%)	
Male	6119 (33.6%)	36 (34.3%)	6083 (33.6%)	
Missing	16	0	16	
**Race/ethnicity**				0.18
Non-Hispanic, White	13733 (75.3%)	78 (74.3%)	13655 (75.3%)	
Non-Hispanic, Black	355 (1.9%)	0 (0.0%)	355 (2.0%)	
Hispanic	2225 (12.2%)	15 (14.3%)	2210 (12.2%)	
Asian	1038 (5.7%)	3 (2.9%)	1035 (5.7%)	
Other	694 (3.8%)	7 (6.7%)	687 (3.8%)	
Missing	195 (1.1%)	2 (1.9%)	193 (1.1%)	
**Body Mass Index**				0.74
Healthy	6817 (38.3%)	42 (40.4%)	6775 (38.3%)	
Obesity	4814 (27.0%)	30 (28.8%)	4784 (27.0%)	
Overweight	5956 (33.4%)	30 (28.8%)	5926 (33.5%)	
Underweight	229 (1.3%)	2 (1.9%)	227 (1.3%)	
Missing	424	1	423	
**Vaccine Type**				< 0.001
Moderna	8662 (47.5%)	34 (32.4%)	8628 (47.6%)	
Pfizer	8273 (45.4%)	46 (43.8%)	8227 (45.4%)	
Johnson & Johnson	1239 (6.8%)	23 (21.9%)	1216 (6.7%)	
Novavax/Other	66 (0.4%)	2 (1.9%)	64 (0.4%)	
**Chronic diseases**				< 0.001
None	7124 (39.1%)	18 (17.1%)	7106 (39.2%)	
One	5397 (29.6%)	27 (25.7%)	5370 (29.6%)	
Two or more	4419 (24.2%)	57 (54.3%)	4362 (24.1%)	
Missing	1300 (7.1%)	3 (2.9%)	1297 (7.2%)	

* Pearson’s Chi-squared.

** Pearson’s Chi-squared test with Yates’ continuity correction.

The prevalence of chronic diseases are as follows: among non-responders, 7.8% reported having asthma, 24.5% cancer, 12.7% cardiovascular disease, 7.8% kidney disease, 2.9% chronic obstructive pulmonary disorder (COPD), 9.8% diabetes or high blood sugar, 29.4% hypertension/high blood pressure, 49% being immunocompromised, 13.7% obesity, and 30.4% reported “other” comorbidities. (Table 2).

**Table 2:** Vaccine responder status versus chronic disease and age-adjusted logistic regression of non-respond status by chronic disease.

	Vaccination Response Status*				Logistic Regression**		
Chronic Disease	Non-Responder (N = 105)	Responder (N = 18135)	Total (N = 18240)	Adjusted-OR (95% CI)		P-value
**Asthma**							
No	94 (92.2%)	14697 (87.3%)	14791 (87.3%)	Ref		Ref
Yes	8 (7.8%)	2141 (12.7%)	2149 (12.7%)	0.61 (0.27, 1.18)		0.18
**Cancer**							
No	77 (75.5%)	15260 (90.6%)	15337 (90.5%)	Ref		Ref
Yes	25 (24.5%)	1578 (9.4%)	1603 (9.5%)	2.59 (1.59, 4.12)		<0.01
**Heart disease**							
No	89 (87.3%)	15852 (94.1%)	15941 (94.1%)	Ref		Ref
Yes	13 (12.7%)	986 (5.9%)	999 (5.9%)	1.76 (0.91, 3.14)		0.072
**Kidney disease**							
No	94 (92.2%)	16615 (98.7%)	16709 (98.6%)	Ref		Ref
Yes	8 (7.8%)	223 (1.3%)	231 (1.4%)	5.11 (2.24, 10.15)		<0.01
**COPD**							
No	99 (97.1%)	16603 (98.6%)	16702 (98.6%)	Ref		Ref
Yes	3 (2.9%)	235 (1.4%)	238 (1.4%)	1.53 (0.37, 4.18)		0.476
**Diabetes**							
No	92 (90.2%)	15340 (91.1%)	15432 (91.1%)	Ref		Ref
Yes	10 (9.8%)	1498 (8.9%)	1508 (8.9%)	0.92 (0.45, 1.70)		0.803
**Hypertension**							
No	72 (70.6%)	12396 (73.6%)	12468 (73.6%)	Ref		Ref
Yes	30 (29.4%)	4442 (26.4%)	4472 (26.4%)	0.90 (0.57, 1.39)		0.632
**Immunocompromised**							
No	52 (51.0%)	15885 (94.3%)	15937 (94.1%)	Ref		Ref
Yes	50 (49.0%)	953 (5.7%)	1003 (5.9%)	15.43 (10.38, 22.9)		<0.01
**Obesity**							
No	88 (86.3%)	14156 (84.1%)	14244 (84.1%)	Ref		Ref
Yes	14 (13.7%)	2682 (15.9%)	2696 (15.9%)	0.83 (0.45, 1.42)		0.523
**Sickle cell disease** ***							
No	102 (100.0%)	16834 (100%)	16936 (100.0%)	-		-
Yes	0 (0.0%)	4 (0.0%)	4 (0.0%)	-		-
**Other chronic disease**							
No	71 (69.6%)	14825 (88.0%)	14896 (87.9%)	Ref		Ref
Yes	31 (30.4%)	2013 (12.0%)	2044 (12.1%)	3.13 (2.02, 4.73)		<0.01
**No chronic disease**							
No	84 (82.4%)	9732 (57.8%)	9816 (57.9%)	Ref		Ref
Yes	18 (17.6%)	7106 (42.2%)	7124 (42.1%)	0.33 (0.19, 0.55)		<0.01
**Number of chronic diseases**							
None	18 (17.1%)	7106 (39.2%)	7124 (39.1%)	Ref		Ref
One	27 (25.7%)	5370 (29.6%)	5397 (29.6%)	1.85 (1.02, 3.44)		0.045
Two or more	57 (54.3%)	4362 (24.1%)	4419 (24.2%)	4.52 (2.65, 8.06)		<0.01

* N-Miss: 3 Non-responders and 1297 responders missing chronic disease information.

** Age adjusted logistic regression: Non-responder ~ Age + Chronic Disease.

*** Insufficient sample size for analysis.

Age-adjusted odds ratios for non-response indicate that immunocompromised participants were 15.43 times more likely to have no response to a vaccine versus those who are not immunocompromised. Participants with kidney disease were 5.11 times and participants with cancer were 2.59 times more likely to have no response to a vaccine versus those without that chronic disease. Participants who reported “other” non-specified comorbidity were 3.13 times more likely to have no response to a vaccine. Furthermore, our results show that participants with two or more chronic diseases were 4.52 times more likely to have no response to a vaccine. Results are shown in Table 2.

Change in the response status for the 105 non-responders was observed in a total of 50 individuals, referred to as the dynamic non-responders group, such that 13 participants tested positive for their Roche S test after 14 days of receiving their PSV with no reported booster doses, 25 participants tested positive for their Roche S test after 14 days of receiving their PSV and one booster dose, and four tested positive following their second booster dose. Eight participants reported a COVID-19 infection within 14–180 days of their PSV and hence tested positive for their following Roche S test after the reported infection. Data are provided in the (S1 Table).

As for the remaining 55 non-responders, referred to as the static non-responders group, the response status did not change even after receiving additional vaccine doses beyond their PSV, as reported on their survey, during the observation window (i.e., the duration of the study) such that: 6 participants completed all four Roche S tests: 3 participants reported receiving two boosters and 3 received one booster. 11 participants completed three tests: 1 received two boosters and 5 received one booster. 14 participants completed two tests; 10 received one booster. The remaining 24 participants completed just one Roche S test, where 1 received one booster. Data are provided in the (S2 Table).

## Discussion

The majority of the results from this population-level survey align with recent literature indicating that those with cancer and pre-existing immune compromising comorbidities have higher odds of vaccine non-response than those without such conditions [1–5]. Our results show that immunocompromised participants were 15.43 times more likely to have no response to a vaccine versus those who were not immunocompromised. This aligns with the systematic review conducted by Galmiche S et al. [7] and the findings of an observational study by Ferri C et al. [6]. Furthermore, our results indicate that participants with cancer were 2.59 times more likely to have no response to a vaccine versus those without cancer. This also aligns with the results found in [1,2].

Our results show that participants with kidney disease were 5.11 times more likely to have no response to a vaccine, and having two or more chronic diseases doubled the odds of vaccine non-response. Our findings suggest additional comorbidities, such as kidney disease and overall chronic disease burden, should be considered (both independently and alongside other chronic diseases and conditions) as risk factors for vaccine non-response.

Recent and previously published studies on this topic also highlighted an urgent need for novel immunization strategies for patients with chronic illnesses [1–7,12]. In addition, some of these studies recommended routine assessment of vaccine antibodies in cancer patients and novel strategies that must be implemented for COVID-19 prevention in these individuals [1,2]. Others suggested a priority for booster doses for patients with suboptimal response, including immunosuppressed individuals or the use of different types of vaccines [6].

As for guidelines and recommendations to efficiently vaccinate individuals with chronic diseases for COVID-19, a comprehensive plan should include the following key components [18–21]; 1. priority should be accorded to individuals with chronic conditions in vaccine distribution phases, taking into account the severity of the condition and its impact on COVID-19 risk; 2. Establishment of specialized vaccination clinics in healthcare facilities that cater to individuals with chronic diseases that ensure accessibility and strict adherence to safety protocols; 3. Collaboration with healthcare providers to identify and promptly inform eligible patients, while delivering precise and consistent information regarding the importance and safety of COVID-19 vaccination; 4. Development of a centralized database for tracking vaccinations, monitoring side effects, and scheduling timely second doses when necessary; and 5. Conducting targeted outreach campaigns tailored to this population to underscore the vaccine’s benefits in reducing COVID-19 complications among those with chronic conditions.

Additional epidemiologic surveillance programs will be helpful to validate further the findings reported here and those observed in smaller samples of vaccinated individuals seeking care for these conditions.

Given health disparities and the large number of individuals in the U.S. diagnosed with one or more chronic illnesses, it is essential to understand vaccine non-response and its impact on the most vulnerable. Given the disproportionate burden of chronic illness in underrepresented and uninsured communities, further assessment of disease burden and vaccine response is needed.

### Limitations

While our study provides important insights, certain limitations should be considered. First, the 90,000 individuals in TX CARES represent a population-level convenience sample, albeit large, demographically diverse, and geographically spread. This means participants choose whether to join the study or not, which can result in selection biases that limit generalizability to the entire population. Second, participants’ data regarding chronic illnesses are self-reported and not verified via Electronic Health Records (EHR). A predefined list of options for chronic diseases is presented in the questionnaire. The options include Asthma, Cancer, Heart disease, Kidney disease, Chronic obstructive pulmonary disease (COPD), Diabetes or high blood sugar, Hypertension/high blood pressure, Immunocompromised, Obesity, Sickle cell disease, Other chronic disease, and No/Not applicable. Participants can select all that apply to their conditions. Since this is self-reported data, some conditions may be under-reported or missed. Third, different medications, illnesses, or conditions result in immunocompromised status; we did not ask for the specific condition that caused a participant to be considered immunocompromised. The specific question we asked was, “Has a doctor or healthcare professional told you that you may have a suppressed or weakened immune system due to a prescribed medication (for example, steroids, methotrexate, etc.), illness, or condition?” This restricted our ability to differentiate between different conditions that can cause immunocompromised status. Fourth, the small number of non-responders did not allow for multivariable analysis. Finally, Texas CARES is a population-based surveillance project to monitor antibody response to SARS-CoV-2 infection and, later in the pandemic, response to vaccination. The questionnaires were developed to minimize patient burden, and many detailed questions regarding treatment options for chronic diseases were intentionally omitted. The questions primarily focused on demographics and COVID-19 recovery rather than clinical care and medical interventions for chronic diseases.

## Conclusions

Consistent with smaller targeted studies on patients with cancer, organ transplant recipients, or those on immune suppressants, this large epidemiologic cohort bears the same conclusion. Furthermore, our findings demonstrate that additional chronic diseases, such as kidney disease and the total number of chronic diseases, are also associated with vaccine non-response. Some remedies that can be considered for reducing the non-response rate are increasing the dose of vaccine shots and/or the frequency of booster shots. These approaches are already implemented in the immunocompromised population; hence, further studies are needed to modify dose adjustment guidelines for COVID-19 vaccines, including people with single or multiple chronic diseases. These chronic conditions should not be restricted to ones directly changing the immune response.

## Supporting information

S1 Table Breakdown of dynamic non-responders (N = 50)*. * Response status change after PSV and/or receiving additional vaccine doses beyond PSV, as reported on the survey, during the observation window (i.e., the duration of the study).
(PDF)

S2 Table Breakdown of static non-responders (N = 55)*. * Response status did not change even after receiving additional vaccine doses beyond PSV, as reported on the survey, during the observation window (i.e., the duration of the study).
(PDF)

S1 Fig Odds ratio and 95% confidence interval and p-value for potential significant factors added to the base models to decide about the adjusted model. To check for the significant factors for our adjusted model, we added each potentially significant factor to our base model and calculated the p-value. Base model: *Non-response ~ all chronic disease*. Base model + predictor: *Non-response ~ all chronic disease + single factor*.
(TIF)

S2 Fig Change in proportions of Spike antibody serostatus across four time points. (TIF)

S3 Fig Visualization of the number of vaccine shots (y-axis), Spike antibody serostatus (vertical lines), and vaccine types (circle color) across time (x-axis) for some of the individuals in the non-responders cohort. (TIF)
