# Supplementary material for: Baseline characteristics of SARS-CoV-2 vaccine non-responders in a large population-based sample
Source: PLoS One. 2024 May 13;19(5):e0303420. doi: 10.1371/journal.pone.0303420 (PMC11090326; doi:10.1371/journal.pone.0303420)
Supplement: S1 Table — * Response status change after PSV and/or receiving additional vaccine doses beyond PSV, as reported on the survey, during the observation window (i.e., the duration of the study). (PDF) [file pone.0303420.s001.pdf]

*S1 Table Breakdown of Dynamic Non-Responders (N=50)**

| Number of participants | Description |
|---|---|
| **13** | Tested positive for their Roche S test after 14 days of receiving their PSV with no booster doses. |
| **25** | Tested positive for their Roche S test after 14 days of receiving their PSV and one booster dose. |
| **4** | Tested positive for their Roche S test after 14 days of receiving their PSV and two booster doses. |
| **8** | Reported a COVID-19 infection within 14-180 days of their PSV and hence tested positive for their following Roche S test. |

* Response status change after PSV and/or receiving additional vaccine doses beyond PSV, as reported on the survey, during the observation window (i.e., the duration of the study)
