# Supplementary material for: Baseline characteristics of SARS-CoV-2 vaccine non-responders in a large population-based sample
Source: PLoS One. 2024 May 13;19(5):e0303420. doi: 10.1371/journal.pone.0303420 (PMC11090326; doi:10.1371/journal.pone.0303420)
Supplement: S2 Table — * Response status did not change even after receiving additional vaccine doses beyond PSV, as reported on the survey, during the observation window (i.e., the duration of the study). (PDF) [file pone.0303420.s002.pdf]

*S2 Table. Breakdown of Static Non-Responders (N=55)**

Number of participants	Description
**6**	Completed all four Roche S tests<br>• 3 participants reported receiving two boosters and<br>• 3 received one booster
**11**	Completed three Roche S tests<br>• 1 received two boosters,<br>• 5 received one booster and<br>• Rest did not report receiving boosters
**14**	Completed two Roche S tests<br>• 10 received one booster and<br>• Rest did not report receiving boosters
**24**	Completed just one Roche S test<br>• 1 participant received one booster and<br>• Rest did not report receiving boosters

* Response status did not change even after receiving additional vaccine doses beyond PSV, as reported on the survey, during the observation window (i.e., the duration of the study)
