# Supplementary figures and images for: Baseline characteristics of SARS-CoV-2 vaccine non-responders in a large population-based sample
Source: PLoS One. 2024 May 13;19(5):e0303420. doi: 10.1371/journal.pone.0303420 (PMC11090326; doi:10.1371/journal.pone.0303420)

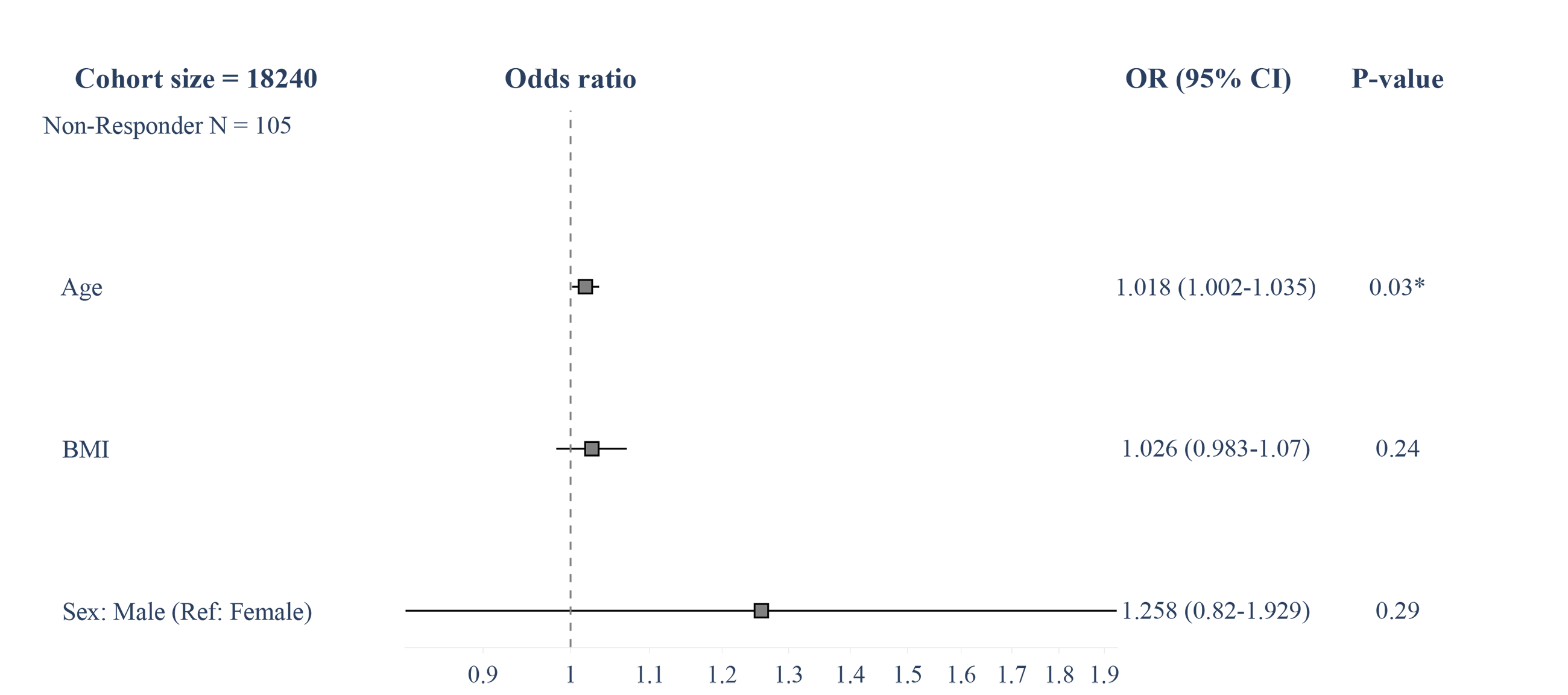

Supplement: S1 Fig — To check for the significant factors for our adjusted model, we added each potentially significant factor to our base model and calculated the p-value. Base model: Non-response ~ all chronic disease. Base model + predictor: Non-response ~ all chronic disease + single factor. (TIF) [file pone.0303420.s003.tif]

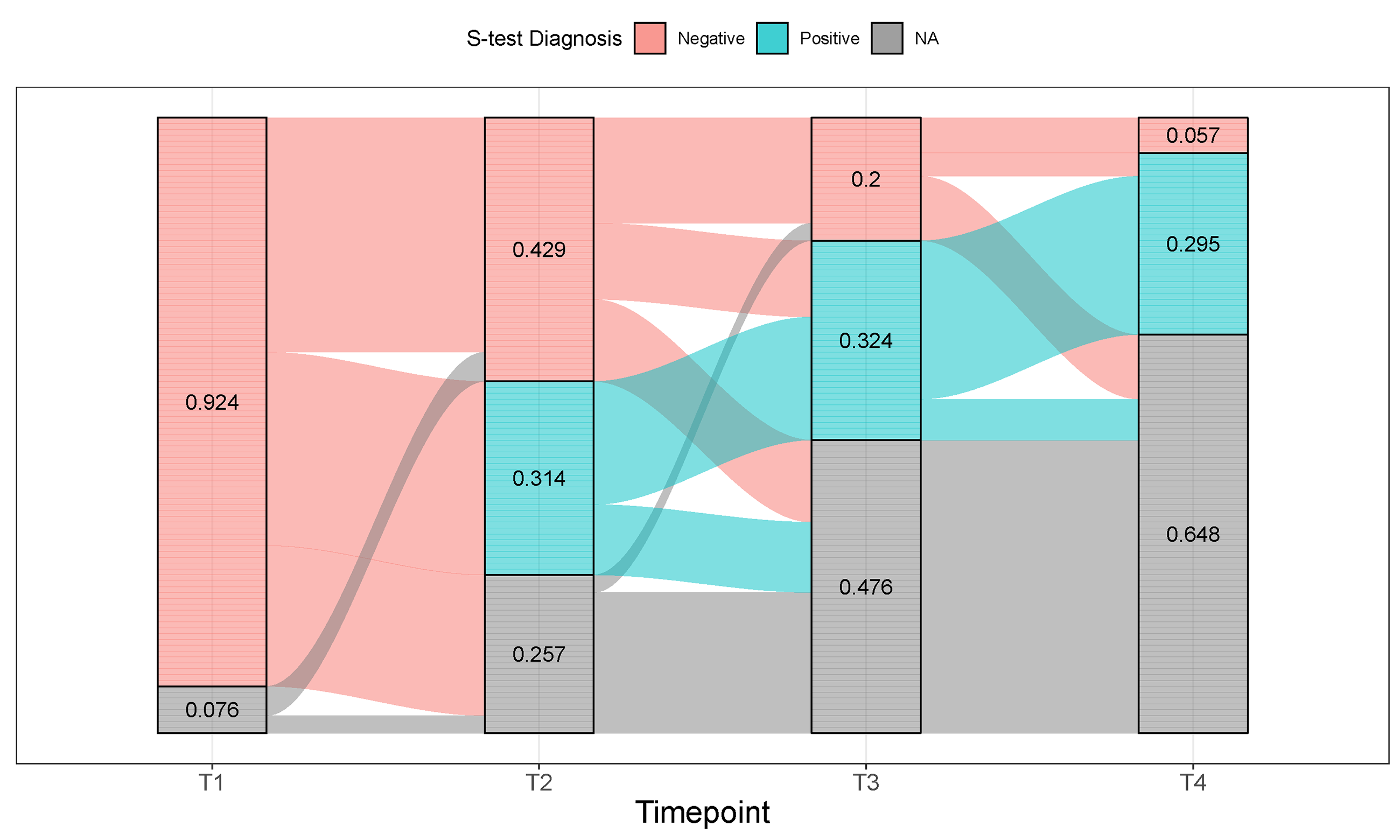

Supplement: S2 Fig — (TIF) [file pone.0303420.s004.tif]

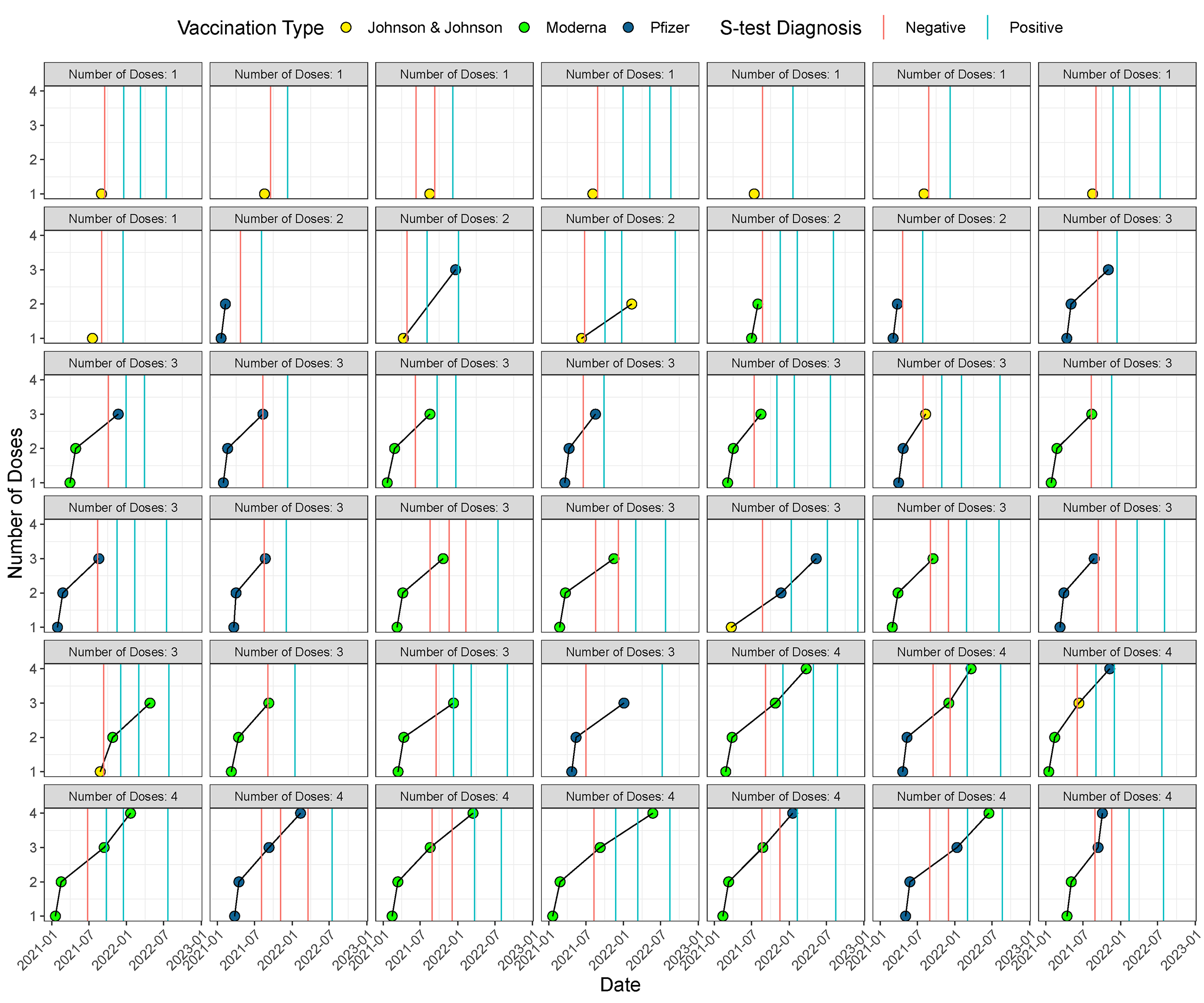

Supplement: S3 Fig — (TIF) [file pone.0303420.s005.tif]
